# Artificial imine reductases: developments and future directions

**DOI:** 10.1039/d0cb00113a

**Published:** 2020-10-16

**Authors:** Rosalind L. Booth, Gideon Grogan, Keith S. Wilson, Anne-Kathrin Duhme-Klair

**Affiliations:** Department of Chemistry, University of York UK anne.duhme-klair@york.ac.uk; York Structural Biology Laboratory, Department of Chemistry, University of York UK

## Abstract

Biocatalytic imine reduction has been a topic of intense research by the artificial metalloenzyme community in recent years. Artificial constructs, together with natural enzymes, have been engineered to produce chiral amines with high enantioselectivity. This review examines the design of the main classes of artificial imine reductases reported thus far and summarises approaches to enhancing their catalytic performance using complementary methods. Examples of utilising these biocatalysts *in vivo* or in multi-enzyme cascades have demonstrated the potential that artIREDs can offer, however, at this time their use in biocatalysis remains limited. This review explores the current scope of artIREDs and the strategies used for catalyst improvement, and examines the potential for artIREDs in the future.

## Abstract 2

This review surveys the development of this class of artificial metalloenzymes and provides a perspective on existing and future research in the area.

## Graphical abstract

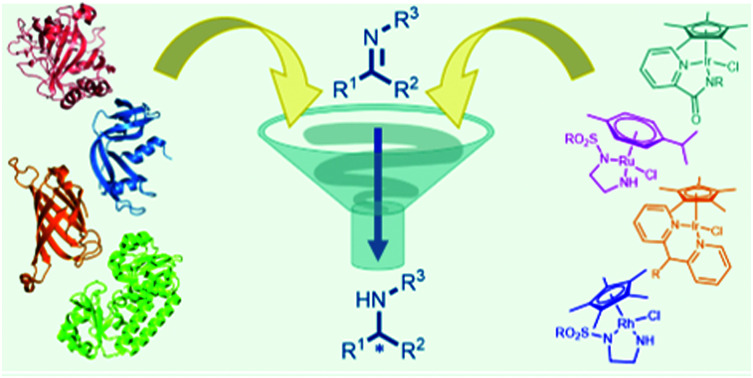

## Introduction

Biocatalysis remains an attractive target, capable of achieving high chemo-, regio- and enantio-selectivities under milder conditions than those required for traditional organometallic catalysis.^1,2^ The preparation of chiral amines is a highly desirable target for biocatalysis. The enantioselective reduction of imines represents one key pathway to achieving some of these intermediates/products. Naturally-occurring enzymes capable of the asymmetric reduction of imine bonds in synthetic substrates were only relatively recently reported by Mitsukura *et al.* in 2010.^4^ Subsequently, the use of bioinformatics to scan large databases of enzymes has led to the discovery and development of many imine reductases (IREDs).^5^ IREDs have been screened for a large number of imine substrates, gradually expanding the known substrate scope of these enzymes. While substantial progress in the field of enzyme engineering, such as the technique of directed evolution, has led to the faster development of other enzymes optimised for target substrates and conditions,^3,6^ with a few notable exceptions,^7,8^ IREDs have received relatively little attention.

*In vitro* evolution has allowed the improvement of some IREDs to enable industrial biocatalysis.^9^ Rational improvement, in contrast, is often hampered by a limited understanding of the determinants of mechanism or stereoselectivity.^5^ It has proven difficult to predict if an IRED is (*R*)- or (*S*)-selective, with some enzymes switching selectivity under different external conditions,^10^ suggesting the determinants of selectivity are extremely complex. Several IREDs have, in addition, been reported to display product inhibition,^11^ a common problem of natural enzymes, which can be solved by feeding strategies, but this requires more specialised experimental set-ups. Other concerns, which include a still limited substrate scope,^5^ poor long-term stability,^12^ high cost of development and the need for expensive cofactors, such as NADH/NADPH^12–14^ suggest that complementary approaches to natural metalloenzyme discovery are merited for imine reduction.

Even before the discovery of natural IREDs, artificial metalloenzymes (ArMs), formed of a reactive metal catalytic centre within a protein scaffold, were designed to target imine reduction.^15^ The thought behind this design is to combine the diverse reaction scope of synthetic organometallic catalysts with selectivity influenced by a secondary coordination sphere from the protein scaffold.^16^ The incorporation of an organometallic complex inside a protein has several potential benefits including (i) compatibility with milder conditions, as for natural enzymes,^17,18^ (ii) protection from inactivation by external factors such as thiols, water and oxygen,^19,20^ (iii) increased rates of catalysis,^21,22^ and (iv) improved enantioselectivity from the presence of an extensive and complex secondary coordination sphere that would otherwise be extremely challenging to achieve synthetically.^23,24^ The organometallic catalysts used for these artificial imine reductases (artIREDs) tend to use formate in place of the more expensive nicotinamide cofactors as a source of hydride for the reduction.^25–27^ The design of ArMs also allows two complementary methods for optimising performance as not only can the steric and electronic properties of the organometallic catalyst be optimised but also the protein scaffold.^28,29^

Although results so far are promising, artIREDs are yet to demonstrate improved selectivity or activity compared to more established chemical reduction methods.^30,31^ The range of accessible products is also limited by the stability of the imine substrates in aqueous conditions necessary for the stability of the protein scaffold. Cyclic imines tend to be stable in such conditions, but more hydrolytically labile noncyclic imines are much less so, limiting the types of amine intermediates that can be produced. Amongst natural enzymes, reductive amination has received more interest compared with imine reduction as it is a method of accessing a wider range of secondary and tertiary amines from cheap and readily accessible ketone and amine precursors.^11^

**Fig. 1:**
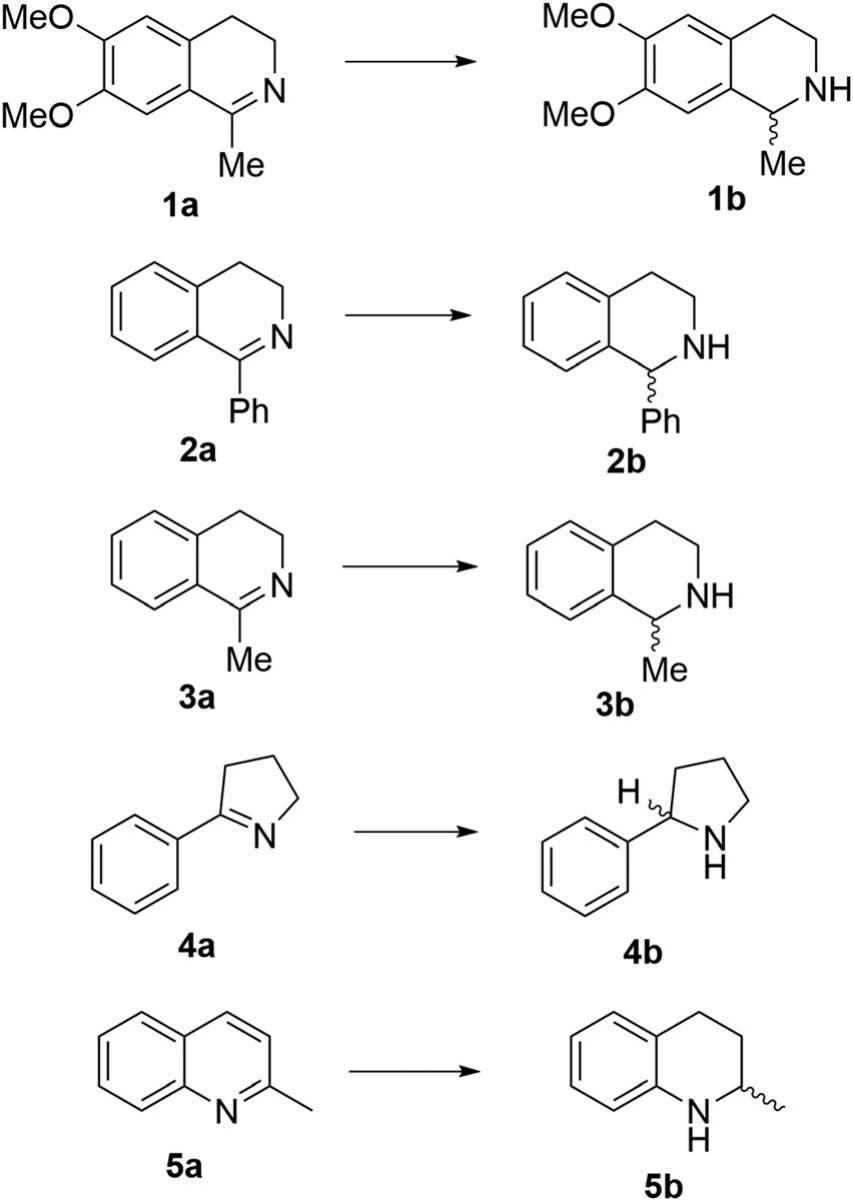
Commercially available substrates for testing the reduction of cyclic imines.

Reports of the use of artIREDs in enzyme cascades, *in vivo* catalysis that removes the need for protein extraction and purification and potential biomedical applications, present exciting future directions for some of these designs. However, the greatest value in this research to date is the contribution to our understanding of the design of ArMs and the development of new methods and techniques for enhancing catalytic performance and selectivity. Much of the work reviewed in this article examines imine reduction as a tool to demonstrate progress, with the reduction of commercially available substrates, such as **1a–5a** (Fig. 1), commonly used as benchmark reactions to examine performance of the artIREDs. In addition, enantiomerically pure salsolidine (**1b**), and other isoquinolines, are valuable as biologically active alkaloids.^32^

There are multiple considerations in designing an artificial metalloenzyme; primarily the selection of a suitable protein. Over 80 different proteins have been used for the construction of ArMs and engineered to catalyse a huge variety of synthetic reactions, covered in a recent comprehensive review^33^ and recorded in an online database (https://amp.ward-lab.ch/), produced and maintained by the Ward group. Likewise, we guide interested readers towards detailed reviews in the areas of natural IREDs^2,10,34^ and synthetic organometallic imine reduction catalysts,^30,31,35^ to avoid covering the same ground here. This review surveys the design of artIREDs and the and evolution strategies employed to improve their performance.

## Scaffolds

### Streptavidin

**Fig. 2:**
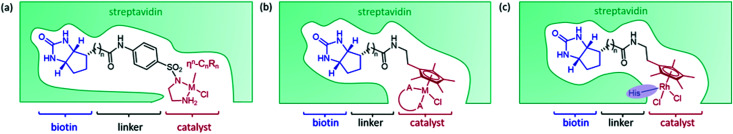
(a) The components of a streptavidin–biotin ArM. The design incorporates biotin, an anchoring unit with a strong affinity for streptavidin. The synthetic catalyst is attached *via* a bidentate ligand to biotin by a linker, *n* = 4 (b) the design for a streptavidin–biotin ArM by which the synthetic catalyst is attached to biotin *via* a linker attached to the arene ligand, *n* = 4 (c) dual-anchoring strategy, *n* = 4.

The streptavidin design exploits the high binding affinity of the protein, derived from *Streptomyces avidinii*, for the small molecule biotin. The non-covalent anchoring strategy is achieved by connecting an organometallic catalyst to biotin by a short linker, either from the bidentate ligand of the catalyst (Fig. 2a)^15^ or from the arene ligand (Fig. 2b).^36^ This cofactor spontaneously binds irreversibly to streptavidin. Dual anchoring has also been attempted with biotin attached to the arene ligand and a coordinating histidine residue incorporated near the site of the metal in the place of either S112 or K121 (Fig. 2c).^37^

Initially, several organometallic transfer hydrogenation catalysts were screened for the reduction of imine **1a**. A η^5^-Cp*Ir piano-stool complex was found to perform best even though the η^6^-(arene)Ru complex had previously proven best for ketone reduction.^28^ This catalyst was then incorporated into the ArM by linking a bidentate ligand of the metal complex to biotin *via* an aryl sulfonamide group. The biotinylated cofactor, which shows no enantioselectivity in the absence of the protein scaffold, when incorporated into wild-type streptavidin (Sav) resulted in a catalyst that furnished the (*R*)-enantiomer of salsolidine, **1b** with 57% e.e.^15^ X-ray crystal structures of several streptavidin (Sav) ArMs revealed that the enantioselectivity is partly derived from the configuration of the metal complex bound in the active site. It was hypothesised that the structure of the protein determined which enantiomer of the synthetic cofactor bound, despite a racemic mixture of the cofactor being added. This design has been extensively investigated, primarily by the Ward group, with successful optimisation strategies achieving high selectivity on a range of trial substrates (Table 1).

**Table 1:** Selection of some of the best enantioselectivities/activities achieved by the artIREDs designs examined in this review. A positive e.e. value reflects selectivity for the (*R*)-enantiomer, while a negative e.e. reflects selectivity for the (*S*)-enantiomer^14,28,30,34,39,45,48^

ArIRED	Substrate														
	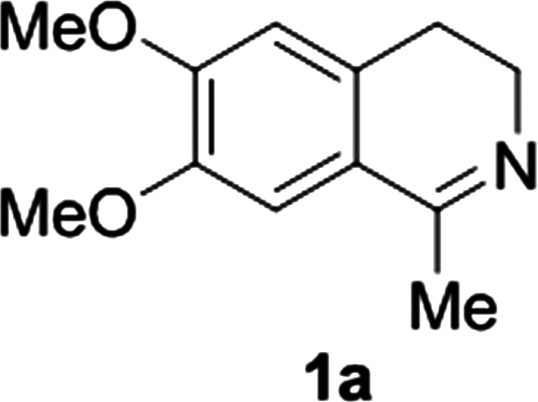			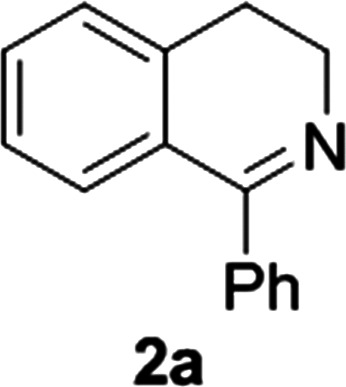			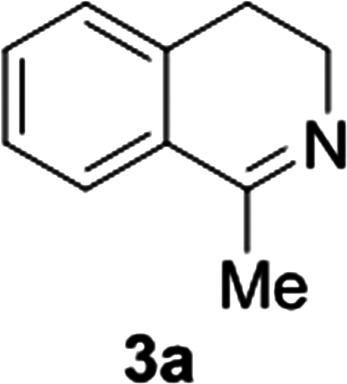			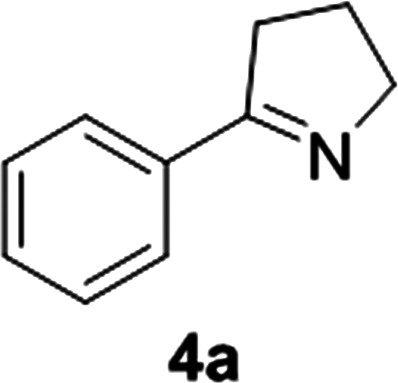			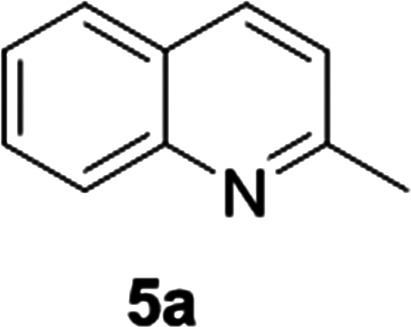		
	Catalyst loading (mol%)	Conv. (%)	e.e. (%)	Catalyst loading (mol%)	Conv. (%)	e.e. (%)	Catalyst loading (mol%)	Conv. (%)	e.e. (%)	Catalyst loading (mol%)	Conv. (%)	e.e. (%)	Catalyst loading (mol%)	Conv. (%)	e.e. (%)
Streptavidin	0.025	Quant.	96	0.5	100	92	0.05	100	91	0.05	99	96			
	1	Quant.	−78	0.5	99	−78	0.25	100	−25				0.5	98	−91
RNase S	0.49	25	−39												
CAII							1	98	74						
	1	98	−94				1	80	−75						
PBP	0.25	100	35												

### Ribonuclease S

**Fig. 3:**
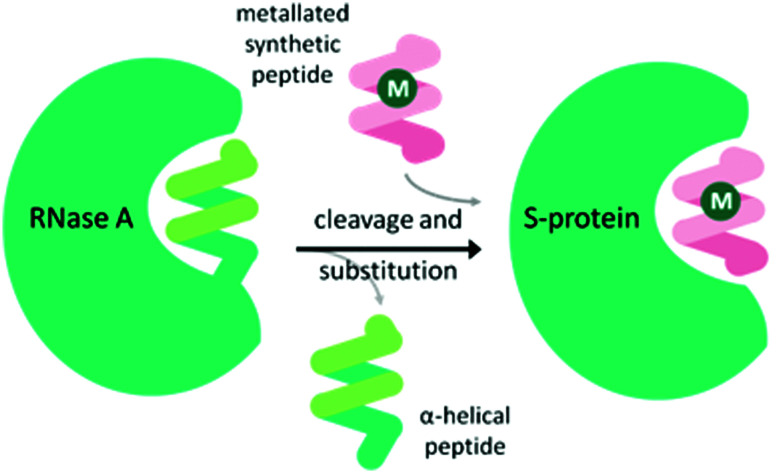
Schematic demonstrating the preparation of the ribonuclease ArM. M = metal ion or organometallic complex.

An α-helical peptide unit can be cleaved from ribonuclease A by subtilisin to leave an S-shaped protein, ribonuclease S, from which the ArM is formed through assembly with a small synthetic α-helical peptide. This non-covalent assembly is achieved spontaneously on combining the S-protein with the synthetic peptide in a buffer solution at 37 °C.^38^ The synthetic peptide can be engineered to include coordinating amino acid linkers, either natural or non-natural, to bind a metal ion or complex. Incorporation of this metallated synthetic peptide with the S-protein results in the formation of the ArM (Fig. 3). Early studies introduced Hg(ii)^39^ and Cu(ii)^40^ ions, but applying this design for a transfer hydrogenase was not attempted until recently.

This design has received only brief attention compared to the streptavidin–biotin model. Multiple transition metal piano-stool complexes were investigated, with IrCp* proving the most successful, with modest rate acceleration on incorporation into the protein scaffold compared to the free metal catalyst. Genetic optimization was attempted *via* rational design where three residues lying in close proximity to the metal ion were selected for mutagenesis, however, the triple mutant afforded no enhanced catalytic performance.^38^ Overall, the best enantiomeric excess achieved was only 39% for (*S*)-**1b** (Table 1).^33^

### Carbonic anhydrase

**Fig. 4:**
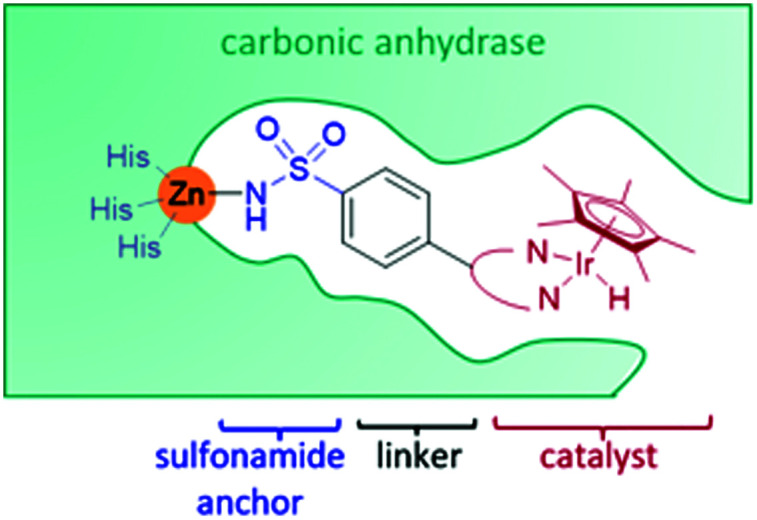
The design of the ArM based on the CAII scaffold, comprising of an aryl sulfonamide anchoring unit which has a strong affinity for a Zn ion, present in CAII. The synthetic catalyst is attached *via* a bidentate ligand to the aryl sulfonamide anchoring unit.

Carbonic anhydrase II (CAII) was selected as a suitable scaffold as it is a stable monomeric protein that can be easily expressed in high yields in *E. coli.*^41^ A Zn(ii) ion lies at the base of a hydrophobic, cone-shaped cavity and has been shown to bind tightly to aryl-sulfonamides.^41,42^ By linking a bidentate ligand to an aryl-sulfonamide group, a Ir-piano stool catalyst was incorporated inside the scaffold (Fig. 4).^42^ To start with, a narrow range of IrCp* organometallic catalysts was incorporated into a small group of CAII variants. The most successful artIRED constructed with wild-type CAII performed the asymmetric transfer hydrogenation **1a** to (*S*)-**1b** at 70% e.e. with a catalyst loading of 8.75 mol%.

### Periplasmic binding protein

**Fig. 5:**
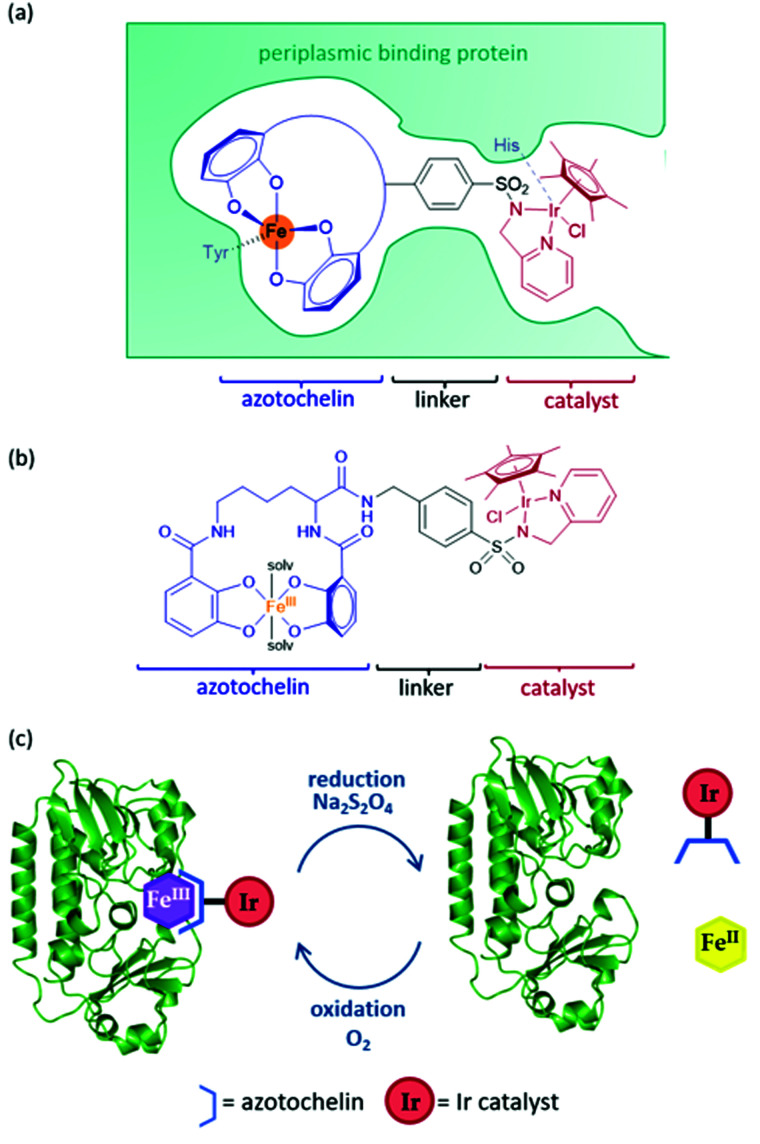
(a) Schematic of the ArM based on a periplasmic protein scaffold. The design comprises of an azotochelin anchoring unit, which forms a complex with Fe^III^, with this complex being bound by the periplasmic binding protein. The synthetic catalyst is attached *via* a bidentate ligand, containing an aryl sulfonamide group, to the azotochelin anchoring unit. (b) Structure of the siderophore-catalyst conjugate, with azotochelin shown in blue, the aryl sulfonamide linker in black and the Ir catalyst sin red. (c) Sketch demonstrating the recyclable nature of the ArM.

An artIRED, designed by the Duhme-Klair group,^43^ takes inspiration from an iron-uptake mechanism of microorganisms that secrete small Fe(iii) chelating molecules called siderophores. The pathway involves the capture of Fe(iii)-siderophore chelates by their cognate periplasmic binding proteins (PBPs) to aid their transport to the inner membrane and into the cytoplasm of the cell. By anchoring an organometallic catalyst to azotochelin, a tetradentate siderophore, this catalyst can be incorporated into the PBP in the presence of Fe(iii) (Fig. 5a).

A key feature of this design is that the binding of this catalyst-siderophore conjugate (Fig. 5b) with the PBP can be reversed by controlling the oxidation state of the Fe cation. While Fe(iii) binds tightly to the siderophore and the complex is strongly bound to the PBP, reduction of Fe(iii) to Fe(ii) results in a less thermodynamically stable and more kinetically labile complex that readily dissociates, releasing Fe(ii).^44,45^ Oxidation of Fe(ii) back to Fe(iii) triggers reassembly of the artIRED without affecting enantioselectivity, allowing recovery and recycling of the costly protein and replacement of the synthetic catalyst in the case of catalyst degradation (Fig. 5c).

Initial work led to a [Cp*Ir(pyridinesulfonamide)Cl] complex that significantly outperformed the more commonly used aminoethylsulfonamide ligand for the reduction of **1a** under mildly acidic conditions tolerated by the protein. The Ir catalyst performed at a rate around 20-fold slower once bound inside the PBP scaffold but produced moderate selectivity for (*R*)-**1b** with an e.e. of 35% (Table 1). The crystal structure revealed that H227 was positioned in close proximity to Ir, indicating a dual anchoring system. This suggested that H227 binds to iridium and may sterically impede the binding of hydride required for the catalytic reaction. While replacement of H227 with non-coordinating alanine did improve the activity of the artIRED, although still not to the level of the free Ir catalyst, it led to a marked drop in enantioselectivity.

## Optimisation and development strategies

The design of ArMs gives two complementary methods for optimising performance, through tuning the organometallic cofactor and by adapting the secondary coordination sphere provided by the protein. There have been multiple approaches to optimising both of these components.

### Optimising the chemical component

The organometallic catalyst can be tuned, both through the metal and the coordinating ligands. Examples of exchanging the type of metal used are usually covered in the initial design of these ArMs. Work by Dürrenberger *et al.*,^15^ which established the streptavidin–biotin design for imine reduction, investigated several catalysts including complexes of ruthenium, rhodium and iridium, with a IrCp* catalyst proving most successful, although arene-linked rhodium complexes have also received attention.^36,37^ Initial work on the Ribonuclease S artIRED also tested complexes of ruthenium, rhodium and iridium, again concluding an IrCp* performs best in this scaffold also.^38^

Tuning the electronic properties of the catalyst *via* the ligands was also investigated in early works optimising the streptavidin, CAII and periplasmic binding protein scaffolds. The streptavidin design was altered with the organometallic catalyst linked to biotin *via* the arene ligand to allow greater freedom to change the bidentate ligand of the iridium catalyst. Ligands containing an amino amide core delivered the highest enantioselectivity for the reduction of **2a** when incorporated into wild-type Sav with up to 67% e.e. for (*S*)-**2b**.^36^ Monnard *et al.*^42^ likewise prepared a range of bidentate ligands for optimising the design of the CAII artIRED, achieving 70% e.e. for (*S*)-**1b** for WT CAII. In devising the PBP artIRED, it was found a [Cp*Ir(pyridinesulfonamide)Cl] complex significantly outperformed the more commonly used aminoethylsulfonamide ligand for the reduction of **1a** hence was the catalyst of choice in this design.^43^

Furthermore, the length and attachment of the linker between the catalyst and the anchoring unit can greatly affect the performance of the catalyst. In one approach, a family of complexes with related structures, but variable linker lengths were incorporated into CAII.^17^ Linkers that were shorter than optimal had a very high dissociation constant, indicating that the biotinylated cofactor readily dissociated from the protein. Cofactors containing linkers one and two atoms longer than this gave much smaller dissociation constants, in line with quantitative binding of the cofactor to CAII, but markedly different catalytic activity, exhibiting the importance of the secondary coordination sphere to which the metal is exposed.

### Optimising the protein scaffold

#### Site-directed and site-saturation mutagenesis

One of the most widely used approaches to optimising the protein is rational design, where residues in close proximity to the metal, identified from crystal structures of ArMs, are exchanged for other amino acids or peptide motifs in an attempt to alter the secondary coordination sphere.^46,47^ The most common changes are exchanging non-coordinating amino acids for coordinating residues or the addition or removal of bulky residues in the binding pocket. The nature of the amino acids in the active site affects the stereochemistry at the metal complex and the position in which the substrate can interact with the metal complex; hence, their alteration provides opportunities for the optimisation and customisation of ArMs.

An interesting example attempted to confine the metal cofactor inside streptavidin by adding sterically bulky structure around the biotin-binding vestibule.^48^ Analysis of crystal structures of Sav artIREDs revealed that the metal ion is not precisely localised in the Sav protein, indicated a by less than full occupancy. It was thought that confining the metal more precisely would improve catalytic activity. Multiple residues around the active site were individually exchanged for short protein loops. Mutants with no change in protein stability or tendency to aggregate were identified and these positions were selected for the insertion of well-defined, naturally occurring protein motifs, as a method of introducing steric bulk. The resulting chimeric scaffolds did not affect biotin-binding capability but nor did they significantly help in localising the metal cofactor, although, in some cases, improved turnover numbers (TONs) were observed.

Site-saturation mutagenesis goes a step further, where selected residues are exchanged with a complete or slightly reduced library of amino acids and the resulting variants screened for improved catalytic activity. This method has an advantage in that it does not require the exact effects of exchanging one amino acid for another to be predicted, screening a library of variants often produces more successful results. For streptavidin, this initially identified two variants, S112A which produced (*R*)-**1b** (96% e.e.) and S112K, which produced (*S*)-**1b** (78% e.e.).^15^ The fact that selectivity for either enantiomer can be accessed through modifying the same protein structure emphasises the importance of the directing effect of the secondary coordination sphere.

#### Computational design

The trial-and-error approach to optimising the protein component of the ArM is time-consuming and costly.^49^ Computational modelling was used to map the mechanistic behaviour of a Sav artIRED to gain a better understanding of how different residues in the secondary coordination shell affects the enantioselectivity.^50^ Computations for two (*R*)- and (*S*)-selective Sav variants revealed that the energy difference between the transition states for the two products agreed well with the observed enantioselectivity. It is hoped that by aiding our understanding of reaction mechanisms, computational modelling can help identify changes to optimise the protein structure directly through rational design, reducing the number of mutants to be tested.

The Rosetta design algorithm was employed by the Baker and Ward groups^29^ in an attempt to optimise the CAII artIRED and demonstrate the progress of computational methods on enzyme redesign. It was theorized that improving the stability and localization of the organometallic cofactor would lead to improved occupancy and performance. The algorithm identified four CAII variants with small numbers of mutations that either increased the hydrophobicity of the binding pocket, improved the packing interactions between the protein and the cofactor, or increased the rigidity of the protein backbone. These mutations led to a 46- to 64-fold improvement in the affinity of the cofactor for the protein scaffold and to significant improvements in enantioselectivity and activity. Modifications to the cofactor to increase the hydrophobicity of the Cp* ligand gave further improvement in enantioselectivity. This resulted in the most (*S*)-selective artIRED for **1b** reported up to 2015, with one of the variants performing with 96% e.e. and a 6-fold increase in turnover number compared to the wild-type artIRED.^33^

#### Engineering to improve fine-tuning

**Fig. 6:**
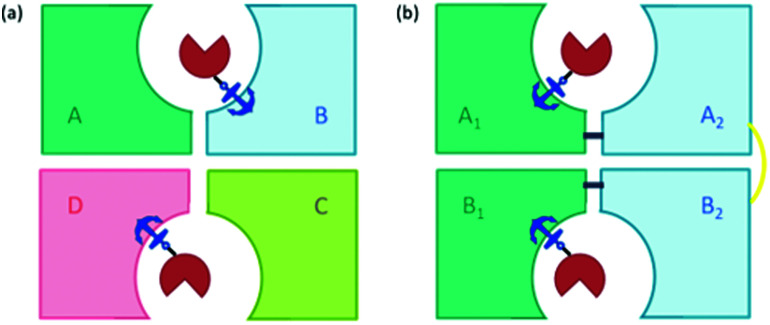
(a) Scheme of streptavidin assembled as four units (tetramer) where the biotin-binding pockets of two neighbouring units are located at a shared interface, showing one biotinylated cofactor bound per dimer. The anchor represents the anchoring group, biotin, red = catalyst. (b) The single chain dimer (scd) design where the two units of each dimer are linked *via* a short peptide chain (navy line) to form dimer A (made up of A_1_ and A_2_) and dimer B (made up of B_1_ and B_2_). These two dimers are linked *via* a disulfide bridge (yellow line) by mutating H127 for cysteine.

A larger scale engineering approach on the streptavidin design was recently reported to improve the fine-tuning of the secondary coordination sphere.^51^ Streptavidin assembles as a tetramer, constructed from a dimer of dimers, where the biotin-binding pockets are located at the interface between two protein chains (Fig. 6a). Any mutation of residues in this pocket is, therefore, displayed twice, reducing the amount of fine control in engineering the protein structure. By fusing the C-terminus of one subunit to the N-terminus of a second *via* a 26 amino acid linker, a single-chain dimer (scd) was formed where mutations can now be selectively made in one subunit without being reflected in the other. Additionally, H127, at the interface between the resulting pair of dimers, was mutated to a cysteine to form a disulfide bridge, further stabilising the structure (Fig. 6b). The resulting finely tuned mutants performed well, with many displaying significant rate enhancement of the Cp*Ir cofactor, as well as good enantioselectivity. The best performing variants on substrates **1–5a** achieved the following significant results: (*R*)-**1b** with 100% conversion and 96% e.e., (*R*)-**3b** with 99% conversion and 93% e.e., and (*R*)-**4b** with 90% conversion and 98% e.e. with catalyst loadings of 0.25 mol%. Substrates **2a** and **5a** proved more challenging, with all variants displaying only moderate enantioselectivity for **2b** and poor conversion to **5b**.^51^ Additionally the biotin-binding capacity of one unit in the single-chain dimer could be removed to reduce the variability of catalytic performance depending on the cofactor : Sav ratio. Most monovalent scdSav artIREDs were found to outperform their equivalent divalent scd artIREDs, among them, the best variants for producing (*R*)-**3b** (91% e.e., 100% conversion, 0.05 mol% catalyst loading), (*R*)-**4b** (96% e.e., 99% conversion, 0.05 mol% catalyst loading) and (*S*)-**5b** (91% e.e., 98% conversion, 0.5 mol% catalyst loading).^51^

#### Directed evolution

Although other design strategies had initially had some success, it has proved challenging to predict the subtle effects of protein structure and bonding on enantioselectivity.^23,52^ To date, our lack of understanding of how the fine details of protein structure relate to activity has resulted in limited progress in the development of biocatalysts by rational design.^22^ Instead, researchers have looked to the combinatorial approaches used in the optimisation of natural enzymes, in particular, directed evolution, which has provided a massive breakthrough in the engineering of enzymes for increased stability, activity, selectivity and reaction scope.^52^ Directed evolution is the use of repeated cycles of random mutagenesis and/or DNA recombination to bring about diversity in the protein scaffold. High-throughput screening methods are essential to identify those protein variants with improved characteristics.

**Fig. 7:**
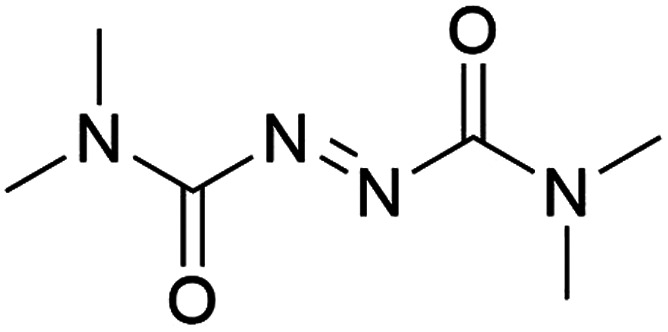
Structure of the diamine DiAm.

It was previously recognised that screening mutants was far too time-consuming to consider using directed evolution because of the need to purify proteins before addition of the biotin-catalyst cofactor. If catalyst testing could be carried out in cell extracts and cell lysates, it would speed up development of artIREDs. However, screening in cellular contents poses problems as heavy metal catalysts are often deactivated by cellular components such as thiols, in particular glutathione. Pre-treatment of cell extracts and lysates with an oxidising agent could be one way of overcoming this limitation since oxidised glutathione does not significantly affect heavy metal catalysts. A diamine, DiAm (Fig. 7), proved most effective, allowing for measurable activity of artIREDs in both cell-free extracts and cell lysate,^53^ which significantly reduces the time needed to prepare multiple variants for screening.

In a pioneering study,^54^ directed evolution was carried out on a small library of amino acids located around the iridium catalyst. It was concluded that bulky residues can limit the degrees of freedom of the bound cofactor, increase the interactions between the protein and the metal ion and influence the enantioselectivity observed. Two mutants, one selective for (*R*)-**2b** (95% e.e.) and one for (*S*)-**2b** (86% e.e.), were identified, with both displaying increased reaction rates and enantioselectivity compared to the previously best performing Sav artIREDs. The study also explored the tolerance of mutants to biphasic conditions, with some mutants showing improved enantioselectivity, although for others reduced activity was seen.

#### Immobilisation and encapsulation

Strategies to improve the stability and performance of Sav artIREDs by use of immobilisation or other tertiary environments have been investigated. Immobilisation of Sav artIRED variants on silica nanoparticles covered with an organosilica film was found to protect the catalysts from deactivation, even in the presence of cellular debris and the organic solvent methanol. The nanoparticles could be recycled up to three times with only a minor loss of selectivity.^12^

Another study encapsulated S112A, S112K and S112A-K121A Sav artIRED variants inside ferritin, an iron storage protein, which provides a tertiary coordination shell for the solvent exposed catalyst. Encapsulation of the artIRED was achieved by exposing ferritin to low pH, at which the protein would unfold, before raising the pH together with the addition of the artIRED, allowing ferritin to refold around the artIRED. This had a remarkable effect on enantioselectivity, with (*S*)-**1b** produced irrespective of the Sav mutant used, proving that this tertiary coordination sphere strongly influences the catalytic mechanism. This inversion of enantioselectivity was not seen, however, for **2b**, with the observed selectivity in line with that expected for the un-encapsulated artIRED. Overall, the encapsulated artIREDs performed relatively poorly with regards to conversion and enantioselectivity but some mutants did display an increased TON.^55^

## Future directions

### Biocatalysis

#### In vivo

**Fig. 8:**
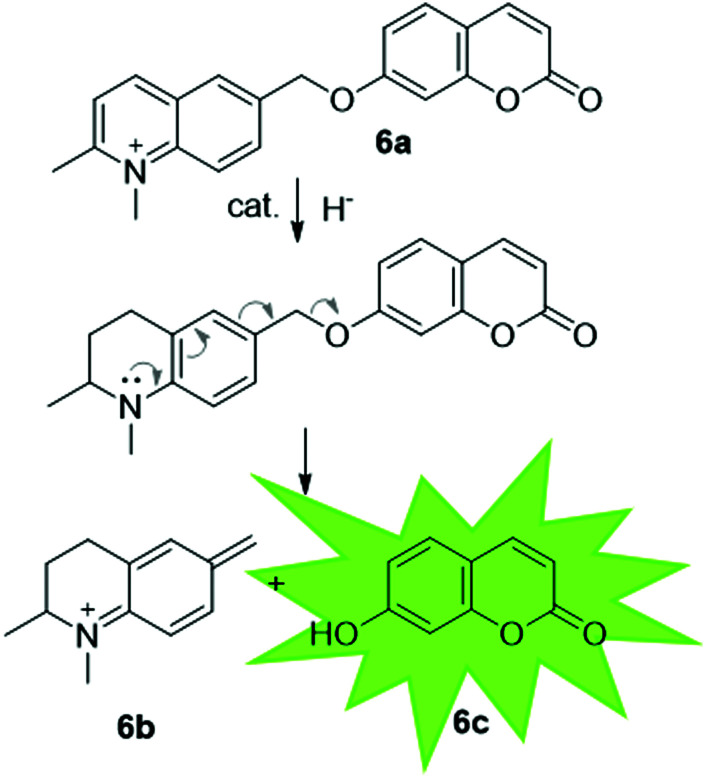
Highly conjugated substrate, which on reduction of the imine, decomposes to release umbelliferone, which can be detected by fluorescence spectroscopy.

*In vivo* catalysis offers an attractive alternative to isolated ArMs since it removes the need for protein extraction and purification steps making them easier to apply to large-scale catalysis. If the engineered ArM is proven to be stable inside the cell, it brings additional benefits of increased protection from external factors and easy removal following catalysis. *In vivo* catalysis has been achieved with Sav artIREDs by engineering Sav to be expressed in the periplasm of *E. coli.*^56^ The periplasm was targeted due to the significantly lower concentration of glutathione compared to the cytoplasm. Glutathione deactivates many organometallic catalysts, even once incorporated into an ArM, hence it was hoped that by expressing Sav in the periplasm, the artIRED could assemble and perform imine reduction successfully. A 24-residue motif with a well-defined tertiary structure was engineered to be incorporated near to the surface of the biotin-binding site in a further attempt to minimise deactivation of the catalyst by external factors. Substrate **6a** was used to test localisation and activity of artIREDs since reduction of the imine bond triggers decomposition to **6b** and the fluorescent dye umbelliferone, **6c** (Fig. 8), allowing the reaction to be followed by fluorescence spectroscopy. Results proved the successful localisation of Sav in the periplasm and the formation of a functional artIRED by addition of biotinylated cofactor.

#### Enzyme cascades

**Fig. 9:**
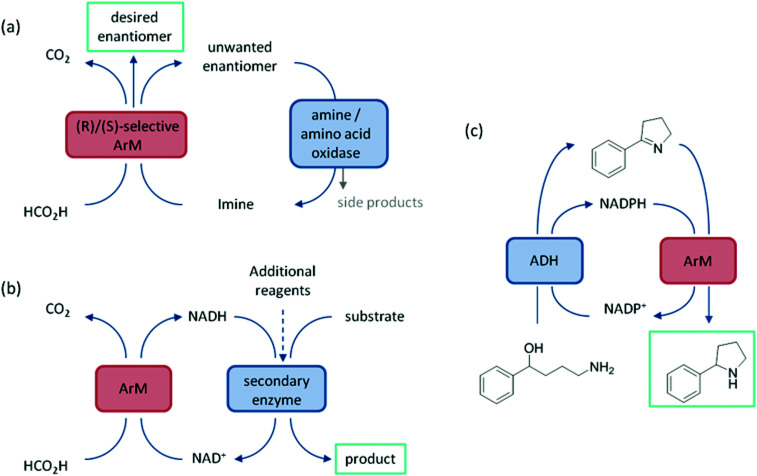
(a) Schematic of the first category of enzyme cascade which lead to the enrichment of one enantiomer from the reduction of the imine by the ArM. (b) A general scheme for the second category of cascade where the role of the ArM is recycling a cofactor and the product of interest is produced by the downstream enzyme. (c) A cascade for a multistep synthesis where the ArM is responsible both for cofactor recycling and producing the end product.

Enzyme cascades represent an exciting application of these ArMs, being an attractive method of achieving complex multistep syntheses without the use of protecting groups or the isolation and purification of intermediates. There have been several successful reports of Sav artIREDs being incorporated into cascades with natural enzymes. Cascades examined to date can be split into two categories, the first being the introduction of artIREDs into cascades where additional enzymes are used to enhance the enantiomeric purity of an amine product from the reduction of an imine substrate by the artIRED (Fig. 9a). These cascades typically consist of a selective artIRED which favours production of one enantiomer of the amine, and a second enzyme, for example a MAO (monoamine oxidase), LAAO (l-selective amino acid oxidase) or DAAO (d-selective amino acid oxidase), which selectively oxidises the unwanted enantiomer back into the imine starting material leading to accumulation of the desired enantiomer over time.^57^

ArtIREDs have also been engineered to use the bioavailable hydride donor cofactors NADPH or NADH in place of formate. While such artIREDs have been combined in the first category,^58^ they have had wider use in the secondary category of cascades, where the product of interest is formed by a downstream natural enzyme (Fig. 9b). Downstream enzymes incorporated so far have performed hydroxylations^57^ and asymmetric reduction of α,β-unsaturated compounds^14^ along with examples of multistep cascades. In one such example, an alcohol dehydrogenase was used to regenerate NADPH. A two-step cascade was designed to be carried out by the alcohol dehydrogenase and the artIRED. In the first step, a linear amino alcohol was converted to cyclic imine **4a** by the alcohol dehydrogenase with simultaneous reduction of NADP^+^. The artIRED then used the resulting NADPH to reduce the imine **4a** to its corresponding amine, **4b** (Fig. 9c).^58^

#### Challenges

There are, however, significant limitations to be solved before artIREDs can be seriously exploited for biocatalysis. So far, few attempts have been made to carry out catalysis on an industrially-relevant scale.^51^ Even then, the access to a limited number of amine products, plus competition from IREDs, may give few biocatalysis options. Attempts to improve stability, tolerance to organic solvents or cheaper costs should lead to improved commercial prospects, however, the engineering techniques that can be used to improve these characteristics are equally applicable to natural enzymes hence artIREDs offer no increased advantage.

### Biomedical applications

It has been proposed that artIREDs could have significant biomedical applications. Carbonic anhydrases are overexpressed on the surface of multiple types of cancer cells. It is hoped that by converting these surface exposed proteins into ArMs, anti-cancer “prodrugs” could be activated in the immediate proximity of cancer cells in the future. In this system, genetic optimisation of the protein was not accessible since it relied on the expression of CAII as it would appear on the surface of host cells, hence a study was carried out to determine the best design of arylsulfonamide-catalyst cofactor. Strains of *E. coli* were engineered to express CAII in localised compartments of the cell; the cytoplasm, periplasm and the cell surface. The selected cofactors were then introduced and the activity of the artIREDs tested on an imine substrate **6a**, which releases the fluorescent dye umbelliferone **6c** on reduction. The results identified an improved cofactor for incorporation into wild-type CAII for *in vivo* whole-cell catalysis.^17^ It will prove exceptionally interesting if the work reported in *E. coli* with CAII artIREDs can be replicated in eukaryotic cells.

Engineering artIREDs to accept nicotinamide cofactors as hydride sources in place of formate potentially opens up further biomedical applications as iridium piano-stool complexes of the type commonly incorporated into the artIRED designs included in this review have been shown to have anti-cancer properties.^59^ Whether artIREDs could have the same effect remains to be seen.

## Conclusions

Over a range of different artIRED designs and optimisation strategies, significant progress in catalytic performance and enantioselectivities has been achieved. The development of new screening techniques has proven important in allowing faster development and use of methods, such as directed evolution.^53,54,56^ Progress in the use of computational modelling to better understand the interactions between the synthetic catalyst and the protein looks to have potential to help with the design of better performing ArMs.^49,60^ Immobilisation of artIREDs has provided additional ways to improve stability and recovery strategies.^12,55^

In summary, artIREDs have exciting potential biomedical applications in addition to biocatalysis opportunities, in particular their use in enzyme cascades and in whole cell *in vivo* catalysis. Successes in altering and improving the activity of artIREDs will play a crucial role in designing and developing ArMs, not only for imine reduction, but also other synthetically challenging reactions. The knowledge gained from optimising ArM designs for imine reduction will help to establish new strategies and methods for producing efficient, highly enantioselective ArMs.

## Conflicts of interest

There are no conflicts to declare.

## Supplementary Material
